# Perioperative use of crystalloids in patients undergoing open radical cystectomy: balanced Ringer’s maleate versus a glucose 5%/potassium-based balanced solution: study protocol for a randomized controlled trial

**DOI:** 10.1186/1745-6215-15-276

**Published:** 2014-07-08

**Authors:** Lukas M Löffel, Bettina Kleeb, Fiona C Burkhard, Patrick Y Wuethrich

**Affiliations:** 1 Department of Anesthesiology and Pain Medicine, Inselspital, Bern University Hospital, CH-3010 Berne, Switzerland; 2 Department of Urology, Inselspital, Bern University Hospital, Bern, Switzerland

**Keywords:** Crystalloid solution, Return of the gastrointestinal function, Radical cystectomy

## Abstract

**Background:**

The optimal crystalloid solution to use perioperatively in patients undergoing open radical cystectomy remains unclear. Many of the fluids used for intravenous hydration contain supraphysiologic concentrations of chloride, which can induce hyperchloremia and metabolic acidosis, resulting in renal vasoconstriction and decreased renal function. In addition, patients receiving less fluid and less sodium show faster recovery of gastrointestinal (GI) function after colonic surgery.

**Methods and design:**

This is an investigator-initiated, single-center, randomized, controlled, parallel group trial with assessor-blinded outcome assessment, in the Department of Urology, University Hospital Bern, Switzerland. The study will involve 44 patients with bladder cancer scheduled for radical cystectomy and urinary diversion. The primary outcome is the duration between the end of surgery and the return of the GI function (first defecation). Secondary outcomes are fluid balance (body weight difference postoperatively versus preoperatively) and the incidence of kidney function disorders according to the Risk – Injury – Failure – Loss - End Stage Renal Disease (RIFLE classification). An equal number of patients are allocated to receive Ringerfundin® solution or a glucose/potassium-based balanced crystalloid solution as baseline infusion during the entire time that intravenous administration of fluid is necessary during the perioperative period. The randomized crystalloid solution is infused at a rate of 1 ml/kg/h until the bladder has been removed, followed by 3 ml/kg/h until the end of surgery. Postoperative hydration is identical in both groups and consists of 1,500 ml of the randomized crystalloid solution per 24 hours. Postoperative patient care is identical in both groups; patients are allowed to drink clear fluids immediately after surgery, and liquid diet is started on postoperative day 1, as well as active mobilization and the use of chewing gum. Body weight is measured daily in the morning. Time of first flatus and first defecation are recorded.

**Discussion:**

This trial assesses the benefits and harms of two different balanced crystalloid solutions for perioperative fluid management in patients undergoing open radical cystectomy with urinary diversion, with regard to return of GI function and effects on postoperative renal function.

**Trial registration:**

Current Controlled Trials ISRCTN32976792 (registered on November 21 2013).

## Background

A recent randomized double-blind clinical trial has demonstrated that intraoperative restrictive hydration with a maleate Ringer’s solution (Ringerfundin®) combined with a concomitant norepinephrine infusion reduces the incidence of postoperative complications and hospitalization time [1]. Patients to whom restrictive hydration was administered were more inclined to have a postoperative zero fluid balance (i.e. weight difference on postoperative day (POD) 1 versus preoperatively was 0) compared with patients in the control group, who had a positive fluid balance of approximately 2 kg on POD 1. A positive postoperative fluid balance is known to be associated with increased postoperative complications [2]. In addition, the intraoperative use of norepinephrine, by minimizing the risk of fluid overload, is associated with a lower incidence of acute kidney damage [3].

Although enough evidence exists to support the rational use of perioperative restrictive hydration, the choice of the optimal crystalloid solution to use remains unclear, and intravenous chloride administration is ubiquitous [4]. Many of the fluids used for intravenous hydration contain supraphysiological concentrations of chloride, which can induce hyperchloremia and metabolic acidosis, resulting in renal vasoconstriction and decreased renal function [5]. The effect of chloride on the kidney is of concern because acute kidney injury has been associated with high mortality, and may require invasive renal replacement therapy [6,7]. After administration of NaCl 0.9%, glucose 5% and Ringer’s lactate solutions in normal subjects, the physiological response is a slow excretion of sodium, which can be impaired by hyperchloremia [8,9]. Because patients undergoing cystectomy are prone to have transient creatinine increase, electrolyte disturbance, and acidosis postoperatively, it would make sense to administer a more physiological intravenous solution to these patients perioperatively. In addition, it has been demonstrated that the influence of salt and water balance on gastrointestinal (GI) recovery after colonic surgery is clinically relevant: patients receiving less fluid and less sodium show faster recovery of GI function, resulting in a shorter hospitalization time [10]. This is of crucial importance in patients undergoing cystectomy because they are at risk for postoperative constipation or ileus.

The aim of this study is to evaluate, in a prospective, single-center, randomized, parallel-group, assessor-blinded study design, the physiology of electrolyte and water homeostasis in patients undergoing open radical cystectomy and urinary diversion, using two different fluid regimens. We hypothesize that an approach with a glucose/potassium-based and reduced chloride crystalloid solution will result in faster return of GI function, reduced hypernatremia and hyperchloremic metabolic acidosis, less frequent hyperosmolality, and a better maintained fluid balance compared with a regimen using a balanced Ringer’s crystalloid solution.

## Method and design

### Ethics approval

The study was approved by the local ethics committee (Kantonale Ethikkommission Bern KEKBE; chairperson: Professor Dr N Tueller; registration number 151/13) on October 22, 2013. This study is conducted in accordance with the Declaration of Helsinki and was prospectively registered on November 21, 2013 at http://www.controlled-trials.com/isrctn with the trial identification number ISRCTN32976792.

### Objectives of the study

The main objectives of this study are 1) to analyze if a positive salt and water balance is associated with a delayed return of GI function; 2) to compare arterial pH, electrolyte (Na, K, Cl, Mg, HPO_4_), glucose, and bicarbonate concentrations at 6 h, 24, 48, 72 and 96 hours postoperatively; 3) to measure sodium and water excretion over the same time period; 4) to study the effect of these crystalloid solutions on plasma osmolality, levels of brain natriuretic peptide (BNP), and plasma concentration of the hormones controlling water and sodium excretion (plasma renin, aldosterone, arginin-vasopressin); and 5) to measure the urine osmolality and concentration of Na, Cl and neutrophil gelatinase-associated lipocalin (NGAL).

### Study endpoints

The primary outcome variable is the postoperative return of GI function after open radical cystectomy, defined as the first defecation and expressed in days.

Secondary outcome variables are postoperative first flatus, positive fluid balance (body weight difference postoperatively versus preoperatively); incidence of kidney function disorders according to the RIFLE classification [11]; difference in pH 24 hours postoperatively (metabolic acidosis defined as hyperchloremia, normal anion gap, low plasma bicarbonate); changes in plasma and urine osmolality during the duration of infusion; incidence of hypernatremia during the duration of infusion; incidence of hyperchloremia during the duration of infusion; incidence of hypokalemia or hyperkalemia during the duration of infusion; changes in plasma renin, aldosterone, arginin-vasopressin, and BNP levels; and changes in NGAL urine values.

### Study design

This is a prospective, single-center, randomized, parallel-group, interventional, assessor-blinded trial conducted at the Department of Urology of the Bern University Hospital, Switzerland. We expect to randomize the first patient in August 2014.

### Blinding and randomization

Assessment of the endpoint data will be performed in a blinded manner. The study nurse responsible for data acquisition will not be allowed to see the content of the crystalloid infusion. Laboratory, defecation, flatus, and body weight data will be assessed electronically in the nurse ward office using the CGM Phoenix Workstation 7-i-PDOS^©^; it will not be assessed in front of the patient.

Randomization will be carried out using a computer-generated list with 11 blocks of 4 patients per block. Allocation will be stored in sealed opaque and numbered envelopes. Patients will be included strictly in numerical order.

### Selection of the participants

Consecutive patients with bladder cancer will be identified and recruited during the preoperative assessment of eligibility for an open radical cystectomy with urinary diversion. Participants fulfilling the inclusion criteria will be asked for their signed informed consent as required by the local ethics committee, and in accordance with the Declaration of Helsinki.

Inclusion criteria are age ≥18 years old, type of surgery (open radical cystectomy with urinary diversion; that is, ileal conduit, orthotopic bladder substitute, catheterizable ileal pouch), American Society of Anesthesiologists (ASA) physical status II and III, and written informed consent.

Exclusion criteria are pregnancy, breast-feeding (which are exclusion criteria for this type of surgery itself), congestive heart failure (New York Heart Association (NYHA) classification ≥3), severe hepatic disease (prothrombin ratio <50%), and significant renal dysfunction (estimated glomerular filtration rate <45 ml/min).

### Time course of the study and collection of the data

#### Admission day

After informed consent, patients undergoing open radical cystectomy with urinary diversion will be randomized, according to the concealed numbered envelopes. Preoperative blood samples will be taken (plasma samples: Na, K, Cl, Mg, HPO_4_, glucose, osmolality, renin, aldosterone, arginine-vasopressin, BNP; urine samples: NGAL, Na, Cl and osmolality) and body weight documented. In addition, preoperative American Society of Anesthesiologists (ASA) physical status score, Charlson comorbidity score, and Glasgow prognostic score will be recorded.

Included patients will be requested to note the time of first flatus and to inform the ward nurses accordingly. During the first 5 postoperative days, patients will stay on the intermediate care unit; this will allow rapid assessment of the first flatus and defecation as patients will be continuously monitored and will have at least one visit by the nurse per hour. Assessment of the first flatus and defecation will be electronically documented (including time of event).

### 6 h postoperatively

Blood samples will be taken (arterial blood gas analysis, plasma samples: Na, K, Cl, Mg, HPO_4_, glucose, osmolality, renin, aldosterone, arginine-vasopressin, BNP) as well as urine samples of NGAL, Na, Cl and osmolality.

### Postoperative day 1

Blood samples (arterial blood gas analysis, plasma samples: Na, K, Cl, Mg, HPO_4_, glucose, osmolality, renin, aldosterone, arginine-vasopressin, BNP) and urine samples (NGAL, Na, Cl and osmolality) will be taken. Body weight, fluid balance (that is, difference between preoperative weight and weight on postoperative day 1), and assessment of flatus and defecation will be documented.

### Postoperative days 2 to 4

On each of these days, blood samples (arterial blood gas analysis, plasma samples: Na, K, Cl, Mg, HPO_4_, glucose, osmolality, renin, aldosterone, arginine-vasopressin, BNP) and urine samples (NGAL, Na, Cl and osmolality) will be taken. Body weight and assessment of flatus and defecation will be documented.

### From postoperative day 5

**Figure 1:**
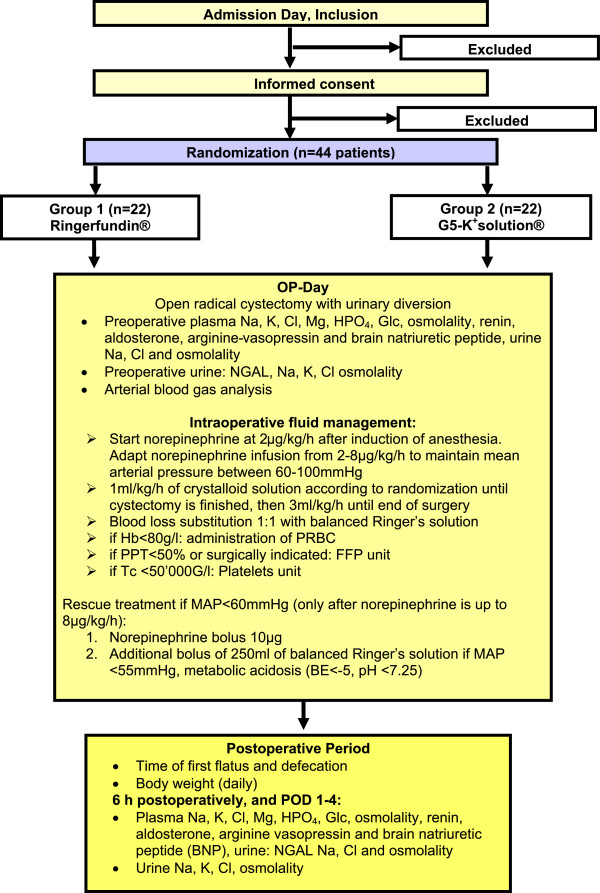
**CONSORT diagram.** BE, base excess; FFP, fresh frozen plasma; MAP, mean arterial pressure; PRBC, packed red blood cell.

Body weight and assessment of flatus and defecation will be documented until return of the GI function (that is, defecation) has occurred.Figure 1 shows the CONSORT diagram of the trial.

### Registration and record-keeping

All data will be collected and recorded onto case report forms (CRFs) by a trained study nurse blinded to the randomization group, which will be recorded onto a secure electronic database in compliance with the new Swiss federal law on research on human subjects (Human Research Act, HFG/LRH) and related ordinances (KlinV/OClin, HFV/ORH, and OV-HFG/Org LRH) and ICH-GCP E6. Data assessment on CRF will be anonymous. In accordance with Swiss law, all original records (CRF and relevant correspondence) will be archived for 15 years in a secure locked room in the Department of Urology, Bern University Hospital, and then destroyed according to the hospital standards concerning destruction of confidential information. This will be carried out even if the trial is terminated prematurely.

### Data monitoring

External, independent monitoring will be performed on site for quality control purposes by a study nurse trained in data monitoring and recording, who will not be involved in the study. This external monitoring will evaluate the progress of the study, and verify the accuracy and completeness of the data recording (CRF). Two monitoring visits will be planned per year. Study data will be centrally monitored by a study nurse (Department of Urology, Bern University Hospital, Switzerland), who is not involved in the recruitment or randomization of patients, or assessment of the data. This study nurse will perform source data verification for all patients, including written informed consent, inclusion and exclusion criteria, and study outcomes.

### Study intervention

An equal number of patients will be randomized to receive either Ringerfundin® solution or G5-K crystalloid solution as baseline infusion during the entire period that an intravenous administration of fluid is necessary (Table 1). The G5-K crystalloid solution is already used as a baseline infusion on our intensive care unit, and has been shown to be safe.

**Table 1:** Electrolyte composition of the two different crystalloid solutions

**Electrolyte**	**Ringerfundin®**	**G5-K solution**
Sodium	145.0 mmol/l	50.0 mmol/l
Potassium	4.0 mmol/l	30.0 mmol/l
Magnesium	1.0 mmol/l	2.0 mmol/l
Calcium	2.5 mmol/l	0 mmol/l
Chloride	127.0 mmol/l	0 mmol/l
Acetate	24.0 mmol/l	0 mmol/l
Maleate	5.0 mmol/l	0 mmol/l
Lactate	0 mmol/l	18 mmol/l
HPO_4_	0 mmol/l	8.0 mmol/l
Glucose	0 mmol/l	50 g/l

### Intraoperatively

After induction of anesthesia, a concomitant norepinephrine infusion will be started at 2 μg/kg/h until the end of surgery, and the randomized crystalloid solution (Ringerfundin®; B Braun Medical AG, Sempach, Switzerland) for group 1 or the G5-K solution for group 2 (Bichsel, Interlaken, Switzerland) will be infused at a rate of 1 ml/kg/h until the bladder has been removed, followed by 3 ml/kg/h until the end of surgery. If hypotension is observed (mean arterial pressure (MAP) <60 mmHg), norepinephrine will be titrated to a maximum of 8 μg/kg/h after an initial bolus of 10 μg. If hypotension persists, a bolus of 250 ml of balanced Ringer’s solution (Ringerfundin®) in both groups will be given.

In both groups, blood loss of >500 ml will be substituted with an equal amount of Ringerfundin®. Packed red blood cells (PRBC) will be transfused if hemoglobin values are less than 80 g/l (<100 g/l in patients with coronary artery disease). Additional boluses of Ringerfundin® (250 ml) will be infused as a rescue medication if MAP persists at less than 60 mmHg after the aforementioned correction, and in cases of severe intraoperative metabolic acidosis (base excess < −5, pH <7.25) attributable to severe hypovolemia.

### Postoperatively

Postoperative hydration will be identical in both groups, and will consist primarily of 1,500 ml of the randomized crystalloid solution per 24 hours [10]. If MAP is less than 60 mmHg after a bolus of 500 ml of Ringerfundin®, norepinephrine will be infused up to a rate of 200 μg/h in both groups. PRBC units will be transfused according to ASA guidelines [12], and fresh frozen plasma units will be given if the prothrombin time is >1.5 times normal value.

In both groups, patients will be allowed to drink clear fluids immediately after surgery while on the intermediate care unit. An oral liquid diet will be started on POD 1 and patients will begin active mobilization. To enhance recovery of bowel function, patients will be treated strictly according to our postoperative enhanced recovery protocol (Table 2). Use of chewing gum will be encouraged [13], and the subcutaneous application of neostigmine 0.5 mg will be started on POD 2 (Table 2). Body weight will be measured daily in the morning. Time of first flatus and first defecation will be recorded.

**Table 2:** **Study relevant information about enhanced recovery program for cystectomy patients at the University Department of Urology, Bern (Cystectomy Enhanced Recovery Approach (CERA**^**©**^**))**

**Time points**	**Interventions**
**Preoperative**	
	No enteral bowel preparation
	Two high enemas the evening before surgery
	Normal nutrition till midnight before surgery
	Clear drinks including carbohydrate till 2 h before surgery
	Subcutaneous injection of low molecular heparin at 20:00 hours
**Intraoperative**	
	DVT prophylaxis with T.E.D.™ hose or sequential compression devices
	Perioperative antibiotics 30 min before surgical incision
	Restrictive fluid regimen aiming at zero postoperative weight gain
	Gastrostomy tube placed, removal of orogastric tube at end of procedure
**Postoperative**	
	DVT prophylaxis with ambulation, T.E.D.™, and subcutaneous low molecular heparin (weight adapted), started 6 hours postoperatively
	Chewing gum encouraged
	Clear drinks allowed the same evening after surgery
	Gastrostomy tube initially left on drainage; closure of the gastrostomy tube will occur when patient is without nausea and vomiting for >24 h
	Bedside mobilization as soon as possible, ideally the same evening after surgery, but if this is not possible, not later than the next morning
	Initial pain treatment with thoracic epidural analgesia, no opioids
	GI ulcer prophylaxis with esomeprazole for at least the first 2 POD
	Antibiotics for 48 hours
	Ambulation on POD 1
	Start oral fluids including energy drinks (Ensure®, Impact®) on POD 1
	Unrestricted clear drinks on POD 1
	Prokinetics: start with 0.5 mg neostigmin subcutaneously up to 4 times per day on POD 2
	Small snacks introduced on POD 2, not later than POD 3
	POD 3: encourage longer mobilization, walking distances, and spending time in a chair
	Anti-emetics given only on request
	Drains removed if draining <50 ml/day
	Gastrostomy tube removed once the patient has passed stool
	POD 5: thoracic epidural removed, oral analgesics (metamizole, paracetamol, hydroxycodon/naloxon (Targin®))

DVT, deep vein thrombosis; POD, postoperative day; T.E.D.™, anti-embolism stockings by Covidien, Mansfield.

### Statistics

Data will be analyzed by the research team in collaboration with a medically versed biostatistician. There will be no interim analysis.

Statistical analysis will be conducted on an intention-to-treat basis. Data distribution will be tested by Q-Q plots. Normally distributed data will be presented as mean and standard deviation (SD), skewed data as median and range, and dichotome data as number and percentage. Comparing related and unrelated continuous samples, the paired and unpaired *t*-tests, respectively, will be used for normally distributed data and the Wilcoxon signed rank test and Mann–Whitney *U*-test, respectively for skewed data. Differences in proportions will be analyzed using the Fisher’s exact test, and risk ratio with associated 95% confidence interval (CI). For the analysis of the primary endpoint, we will use the unpaired Student *t*-test with associated 95% CI. In addition, a multiple logistic regression analysis will be carried out, including the randomization groups and all clinically relevant variables (age, ASA scores, Charlson comorbidity score, Glasgow prognostic score, postoperative fluid balance). A two-sided *P* value of less than 0.05 will be considered significant.

### Sample analysis

Based on retrospective data from our institution in the same surgical population (mean duration until first defecation 4.82 days, SD of 0.82 days), a power analysis was performed using duration of return of GI function as the primary variable. For a calculated sample size of 18 patients in each group (36 patients in total) the study is designed to have an 90% power (β = 0.10) for the Wilcoxon/Mann–Whitney test to detect a difference of 1 day between the groups (Ringerfundin® and G5-K solution) at a two-sided significance level of 5% (α = 0.05) assuming an SD of 1 day (G Power Version 3.1.5., Kiel and Duesseldorf, Germany). This decrease of 1 day is considered clinically relevant. Assuming a drop out of 20%, 22 patients per group will be recruited.

### Assessment of safety

The methods used to measure plasma and urine samples are those established at our hospital and used in everyday clinical practice. Both intravenous solutions will be provided by the pharmacy of the University Hospital Bern. Both solutions are routinely used at our institution (Department of Anesthesia and Department of Intensive Care Medicine).

### Reporting of adverse events

Follow-up will be guaranteed because every patient included in the trial will receive daily visits from the urologist in charge and will have blood samples taken. In addition, all patients will be continuously monitored during POD 1 to 5, as they will be on the intermediate care unit.

The principal investigator (PYW) will monitor the patients for any adverse events (AEs), which are defined as any unfavorable and unintended sign (including abnormal laboratory results), symptom, or disease temporally associated with the use of the randomized infusions. This will be documented on the CRF and immediately communicated to the principal investigator.

Serious AEs (SAEs) are defined as any untoward medical occurrence at any dose that results in death, or is life-threatening (i.e. patient was at risk of death at the time of the event), requires inpatient hospitalization or prolongation of existing hospitalization, or results in persistent or significant disability/incapacity. All treatment related to SAEs will be recorded and reported to the local ethics committee. An investigation will be conducted, and a report on the findings generated before the study can be resumed, in accordance with the Good Clinical Practice Guidelines of the Declaration of Helsinki.

### Duration and timeline

The annual caseload of cystectomy is around 100 in our institution per year. We expect to recruit our participants within 18 months. The study protocol was designed and approval from the local ethics committee obtained in 2013, and participants will be recruited during 2014 and 2015.

## Discussion

The present study will improve knowledge on the use of perioperative crystalloid solutions in patients with bladder cancer undergoing radical cystectomy with urinary diversion. This information should produce a basis on which to develop strategies that aim to prevent perioperative morbidity such as delayed return of the GI function and postoperative renal dysfunction.

The primary outcome of this study is the return of GI function, which is a cornerstone of the postoperative management of patients after major abdominal surgery. Delayed return of GI function is one of the major reasons for prolonged hospitalization time. The potential implications of our results are important. Accelerating the return of GI function will inevitably shorten hospitalization time and thus reduce the cost of hospital expenditure.

We expect by using the G5-K solution, which does not contain chloride anions, to achieve not only a near-zero water balance, as we already demonstrated in a previous RCT, but also a near-zero salt balance after radical cystectomy with urinary diversion [1]. In contrast we expect the patients in the Ringerfundin® group to be at higher risk of having a positive fluid and salt balance. This non-accumulation of salts and water in the G5-K solution group is expected to result in an earlier return of the GI function. Lobo *et al*. already showed that a positive salt and water balance after colonic resection delayed the return of GI function and resulted in longer hospitalization time. In addition, perioperative chloride loading is known to impair urinary output, and to induce metabolic acidosis and even hyperkalemia in patients with preoperative renal dysfunction.

## Conclusion

In conclusion, this trial is an investigator-initiated, pragmatic, randomized clinical trial to test the hypothesis that administration of glucose-rich balanced crystalloid solution containing potassium can accelerate the return of GI function. In addition, this study will also determine the effect of this solution on renal function after radical cystectomy with urinary diversion.

## Trial status

The trial is ongoing.

## Abbreviations

ASA: American Society of Anesthesiologists; BNP: Brain natriuretic peptide; CI: confidence interval; CRF: case report form; GI: gastrointestinal; MAP: Mean arterial pressure; NGAL: Neutrophil gelatinase-associated lipocalin; PRBC: Packed red blood cells; POD: postoperative day; RCT: Randomized clinical trial; RIFLE: risk, injury, failure, loss, end-stage renal disease; SD: standard deviation.

## Competing interests

The authors declare that to have no competing interests.

## Authors’ contributions

PYW/LML/BK drafted the manuscript together with FCB. Study designed by all authors. Logistic organization will be supported by PYW and FCB. All authors read and approved the final manuscript.
