# Erratum to: ‘Perioperative use of crystalloids in patients undergoing open radical cystectomy: balanced Ringer’s maleate versus a glucose 5 %/potassium-based balanced solution: study protocol for a randomized controlled trial’

**DOI:** 10.1186/s13063-016-1221-8

**Published:** 2016-02-18

**Authors:** Lukas M. Löffel, Bettina Kleeb, Fiona C. Burkhard, Patrick Y. Wuethrich

**Affiliations:** Department of Anesthesiology and Pain Medicine, Inselspital, Bern University Hospital, Berne, CH-3010 Switzerland; Department of Urology, Inselspital, Bern University Hospital, Bern, Switzerland

Unfortunately, the original version of this article [1] contained a few errors in Table 1. The correct data for Table 1 has now been included below.

**Table 1:** Electrolyte composition of the 2 different crystalloid solutions

	Ringerfundin®	G5-K Solution®
Natrium	145.0 mmol/l	50.0 mmol/l
Kalium	4.0 mmol/l	30.0 mmol/l
Magnesium	1.0 mmol/l	2.0 mmol/l
Calcium	2.5 mmol/l	0 mmol/l
Chlorid	127.0 mmol/l	65 mmol/l
Acetat	24.0 mmol/l	0 mmol/l
Maleat acid	5.0 mmol/l	0 mmol/l
Lactate	0 mmol/l	18 mmol/l
HPO_4_	0 mmol/l	8.0 mmol/l
Glucose	0 g/l	50 g/l
